# Seizures Temporally Associated With SSRI Treatment: A Two‐Case Report Exploring Agomelatine as a Treatment Alternative

**DOI:** 10.1155/crps/9178274

**Published:** 2026-07-25

**Authors:** José Alfonso Ontiveros Sanchez De La Barquera, Luis Alberto De La Garza Garcia, Guillermo Sanchez Torres, Leopoldo Iván Santacruz Torres, Arturo Alejandro Vera Vidaurri

**Affiliations:** ^1^ Department of Psychiatry, “Dr Jose Eleuterio Gonzalez” University Hospital, UANL, Monterrey, Nuevo Leon, Mexico, uanl.mx; ^2^ Department of Psychiatry, “Tecnológico of Monterrey”, Monterrey, Nuevo Leon, Mexico, udem.edu.mx; ^3^ Department of Infectology, “Angeles Lindavista” Hospital, Mexico City, Mexico

**Keywords:** adverse event, agomelatine, case report, generalized anxiety disorder, major depressive disorder, seizures, SSRIs

## Abstract

Selective serotonin reuptake inhibitors (SSRIs) are widely used as first‐line treatments for major depressive disorder (MDD) and anxiety disorders, with common adverse effects, including sexual dysfunction, anxiety, insomnia, and appetite changes, although seizures have also been reported, particularly in the presence of risk factors such as older age, family history of epilepsy, neurological comorbidities, and high antidepressant dosages. We describe two patients who developed generalized tonic–clonic seizures associated with SSRI therapy. The first case is a man with SSRI‐resistant MDD and multiple medical comorbidities who experienced five generalized tonic–clonic seizures while receiving various antidepressants. The second case is a woman with generalized anxiety disorder (GAD) who developed two generalized tonic–clonic seizures temporally related to paroxetine use. In both cases, extensive investigations, including laboratory tests, electroencephalogram (EEG), CT, and MRI, yielded normal results, suggesting a potential drug‐associated etiology. Both patients were subsequently switched to the atypical antidepressant agomelatine, which was followed by a complete cessation of seizures and marked improvement in mood and anxiety symptoms. These observations underscore the need to consider seizures as a potential adverse event of SSRIs and suggest that agomelatine could be considered an alternative pharmacological option for patients at risk of antidepressant‐associated seizures.

## 1. Introduction

Drug‐induced seizures are a well‐documented side effect of several medications that can lower the seizure threshold. These seizures are clinically similar to those seen in epilepsy [1]. Although focal seizures have been reported, most drug‐induced seizures present as generalized tonic–clonic events and occur when a transient factor temporarily lowers the seizure threshold in an otherwise normal brain [2]. Most drug‐induced seizures are self‐limited and do not lead to long‐term consequences; however, ~9% may progress to status epilepticus, particularly in cases of overdose [3].

Antidepressants at therapeutic doses can lower the seizure threshold, and overdoses may induce seizures [4]. The exact risk is still unclear. Notably, clomipramine and bupropion have been observed to induce seizures even at therapeutic doses [5, 6]. Tricyclic antidepressants (TCAs) are associated with a higher risk, while selective serotonin reuptake inhibitors (SSRIs) are generally considered to have a lower risk of inducing seizures. Observational studies and cases of intoxication suggest that rapid dose escalation or excessively high doses are linked to an increased risk of seizures [4].

In a meta‐analysis that included animal studies, case reports (including overdose cases), and observational data, researchers categorized the seizure risk associated with various antidepressants [7]. Clomipramine, quetiapine, amitriptyline, venlafaxine, citalopram, sertraline, trazodone, mirtazapine, paroxetine, bupropion, and escitalopram were identified as having a moderate risk of inducing seizures. In contrast, fluoxetine and duloxetine were assessed as posing a low risk. Additionally, there was insufficient data available for desipramine, moclobemide, and trimipramine. Meanwhile, lithium, nortriptyline, imipramine, doxepin, tranylcypromine, selegiline, agomelatine, and vortioxetine were categorized as having insufficient data as well but are likely to present a low or very low risk of seizures [7].

Alternative treatment guidelines for patients with psychiatric disorders who are prone to develop seizures with antidepressant treatment are needed. Agomelatine, a melatonergic agent that acts as an antagonist at both 5HT2C and MT1/MT2 receptors, has shown not only antidepressant efficacy but also a favorable profile in the treatment of generalized anxiety disorder (GAD) [8]. The specific psychopharmacological profile of this medication offers a dual mechanism of action. First, it acts as a melatonergic agonist, which helps resynchronizethe circadian rhythms. Second, its role as a 5‐HT2C antagonist increases the release of noradrenaline and dopamine in the frontal cortex. This combination contributes to its effectiveness in treating mood disorders and anxiety while avoiding the typical serotonergic burden that may lower the seizure threshold. This distinctive neurobiological safety profile presents it as a highly suitable option for addressing complex clinical scenarios [9, 10].

In this article, we report two cases, one with resistant major depressive disorder (MDD) and the other with GAD, who developed repeated seizures associated with SSRIs treatment and were successfully treated with agomelatine. We retrospectively applied the Naranjo Adverse Drug Reaction Probability Scale to objectively assess the likelihood of an adverse drug reaction. We suggest that agomelatine could be a promising alternative for patients prone to seizures temporally associated with specific serotonergic antidepressants.

## 2. Case 1

A 41‐year‐old male infectious disease physician, married, and father of two, was referred to our service. There was no family history of psychiatric or neurological disorders. Five years earlier, he had an episode of alcohol abuse due to work‐related stress, but he did not develop a dependence. It is important to note that his first reported seizure occurred about a year later, at the age of 36, indicating that alcohol withdrawal was not the triggering factor for this initial seizure. He had type 1 diabetes mellitus diagnosed at the age of 15, treated with insulin glargine with good adherence under strict medical supervision; he had had one previous episode of diabetic ketoacidosis. Two years before presentation, he was diagnosed with pulmonary tuberculosis and underwent a segmentectomy. Postoperatively, he received a 12‐month DOTS regimen (rifampicin, pyrazinamide, ethambutol, and isoniazid). After surgery, tramadol was prescribed for postoperative pain, and its discontinuation was followed by a difficult withdrawal syndrome that lasted at least 3 months.

### 2.1. Seizure Events During Treatment With Fluoxetine and Vortioxetine

The patient had experienced his first major depressive episode at age 25, which was treated with an SSRI (specific agent not recorded), with remission after several months. A second depressive episode occurred at age 35, characterized by progressive sadness, anhedonia, decreased appetite, insomnia, fatigue, poor concentration, suicidal ideation, and feelings of guilt. Fluoxetine 20 mg/day was initiated. Despite treatment, he remained severely depressed and experienced his first generalized tonic–clonic seizure while driving, which resulted in a car accident. He was admitted to the emergency department, where a mild brain contusion was diagnosed. Laboratory tests and brain MRI were unremarkable, and an electroencephalogram (EEG) showed no epileptiform activity. Hypoglycemia was initially considered as a possible trigger, although blood glucose levels were within the normal range on admission.

Fluoxetine was discontinued, and vortioxetine 10 mg/day was started, which was well tolerated and led to partial improvement of depressive symptoms. However, he subsequently had a second generalized tonic–clonic seizure, requiring a 3‐day hospitalization. Extensive medical, cardiological, and neurological evaluations yielded normal results. Again, hypoglycemia was suspected, although the patient reported careful glycemic control. He continued vortioxetine 10 mg/day plus clonazepam for 1 year, with a good antidepressant response and no further seizures during this period.

### 2.2. Seizure Events During Treatment With Sertraline and Mirtazapine

The patient remained without antidepressant treatment for at least 1 year. At age 38, he developed a third depressive episode. Sertraline was initiated and titrated up to 100 mg/day; due to suboptimal response, mirtazapine 15 mg/day and later olanzapine 5 mg/day were added. Clonazepam 2 mg/day was reintroduced to manage insomnia and severe anxiety. During this treatment regimen, he experienced a third generalized tonic–clonic seizure lasting ~3 min, with a fall from standing height and postictal amnesia, followed by slow recovery. He sustained a fracture–dislocation of the left shoulder that required emergency surgical repair. Laboratory tests and cranial CT were normal, and antiseizure medication was not started at that time.

Two weeks later, while having dinner, he had a fourth generalized tonic–clonic seizure lasting ~3–5 min, accompanied by loss of sphincter control, tongue biting, and postictal amnesia. He was hospitalized again. A Holter study revealed complete third‐degree atrioventricular block, and a pacemaker was implanted. Medical treatment was adjusted to include rosuvastatin 20 mg/day and ivabradine 15 mg/day. The episode of loss of consciousness with convulsive movements was interpreted as being related, at least in part, to the atrioventricular block. EEG and neurological examinations were normal.

While working at the hospital, he experienced a fifth generalized tonic–clonic seizure with a fall and postictal amnesia. On this occasion, the event was associated with diabetic ketoacidosis, and he remained hospitalized until metabolic stabilization was achieved. During the same admission, he had a sixth seizure with similar characteristics. A neurologist then initiated phenytoin. Head CT and EEG again showed no epileptiform activity, rapid rhythms, or beta spindles. He was discharged on phenytoin every 8 h, which was switched 1 week later to levetiracetam 500 mg twice daily. At that time, he continued sertraline 100 mg/day, mirtazapine 15 mg/day, clonazepam 2 mg/day, and olanzapine 5 mg/day, as well as weekly psychotherapy initiated 1 year earlier, with no meaningful improvement in depressive symptoms.

### 2.3. Assessment at Our Clinic and Switch to Agomelatine

The patient was referred to our clinic for further evaluation. He met the criteria for treatment‐resistant MDD. He presented with severe depression characterized by persistent low mood, insomnia, episodes of anxiety, loss of appetite with a 6‐kg weight loss, fatigue, difficulty concentrating, feelings of guilt, and suicidal ideation. These symptoms markedly interfered with his social, family, and professional functioning; he reported missing work several days per week due to profound demotivation. At baseline, the Montgomery–Åsberg Depression Rating Scale (MADRS) [11] score was 27, and the Clinical Global Impression–Severity (CGI‐S) [12] score was 5 (marked depression).

A gradual change in pharmacological management was agreed upon. Agomelatine was introduced at 25 mg/day and increased to 50 mg/day, while other antidepressants were progressively tapered and discontinued. Clonazepam 2 mg/day was maintained. After 3 weeks of agomelatine treatment, he reported an improvement in depressive symptoms, with a reduction of the MADRS score to 15 and a CGI‐S score of 4 (moderate illness). Due to tremor, levetiracetam was reduced to 250 mg twice daily. After 4 weeks on agomelatine, he reported remission of depressive symptoms, with a MADRS score of 6 and a CGI‐S score of 1 (no depression). Over 1 year of follow‐up, there were no further seizure events, and his depression remained in remission. Considering the patient’s history of multidrug treatment for tuberculosis and the known hepatotoxic potential of agomelatine, baseline and regular follow‐up liver function tests (LFTs) were closely monitored. These tests remained within normal limits throughout the treatment. No adverse effects or abnormal laboratory findings related to agomelatine were observed. To objectively assess the likelihood of an adverse drug reaction, the Naranjo scale was applied retrospectively, yielding a score of 4, which indicates a “Possible” adverse drug reaction.

In summary, this patient, who had received several antidepressants, including fluoxetine, vortioxetine, sertraline, and mirtazapine, experienced six generalized tonic–clonic seizures. Laboratory tests, including LFTs, neuroimaging, and EEGs, were consistently normal, and only one seizure was clearly associated with diabetic ketoacidosis. Delays in recognizing a potential association with antidepressant treatment and the absence of early suspicion of drug‐related seizures contributed to significant morbidity, including a car accidents, falls, bone fractures, and multiple hospitalizations. After 24 months of follow‐up, the prognosis regarding seizures occurring during SSRI treatment was considered favorable. The patient remains aware of having had multiple generalized events and continues regular neurological follow‐up. Agomelatine was identified as both a safer antidepressant option and an effective treatment for his resistant MDD. The patient provided written informed consent for the publication of his clinical data.

## 3. Case 2

A 64‐year‐old married woman, a housewife and mother of three with a high‐school education, was referred to our clinic. She had been treated for hypertension with telmisartan and amlodipine for the previous 5 years and for hypothyroidism with levothyroxine following thyroid ablation for hyperthyroidism at age 38. She had a history of breast cancer diagnosed at age 40, currently in remission. Since age 30, she had suffered from irritable bowel symptoms consistent with irritative colitis. She had smoked ~15 cigarettes/day since adolescence and consumed alcohol socially. Her family psychiatric history was notable for a father with MDD; there was no family history of neurological disease.

She first developed symptoms of GAD at age 35 and was treated with paroxetine 20 mg/day plus clonazepam 0.5 mg at bedtime for 6 months, with moderate improvement. At age 55, her anxiety symptoms worsened, prompting reinitiation of paroxetine and clonazepam. Due to insufficient response, olanzapine 5 mg at bedtime was added but discontinued after 1 month because of adverse effects, including weight gain, ataxia, dysarthria, and tics. Paroxetine was increased to 40 mg/day, and valproic acid 500 mg at bedtime was introduced to address treatment resistance.

The patient presented to our clinic requesting medication discontinuation due to persistent daytime drowsiness. A diagnosis of GAD was confirmed using the Mini International Neuropsychiatric Interview (M.I.N.I.) [13] and DSM‐5 criteria [14]. At that time, the GAD 7‐item scale (GAD‐7) [15] score was 0. With the patient, a gradual taper and discontinuation of valproic acid was done. Three weeks after complete withdrawal, she experienced a generalized tonic–clonic seizure witnessed by her husband, characterized by bilateral tonic–clonic movements, followed by complete amnesia for the event. She consulted her general practitioner, but no etiological explanation was provided.

Paroxetine was then slowly reduced from 40 to 20 mg/day, while clonazepam was maintained. Three weeks later, she was admitted to the hospital because of chest pain; a rib fracture was diagnosed. At that moment, she was unable to recall how the trauma had occurred and reported awakening with a headache, multiple bruises, and complete amnesia for the day preceding the onset of thoracic pain, suggesting an unwitnessed seizure followed by a fall. She remained hospitalized for 3 days. Cardiological and neurological evaluations, including EEG and brain MRI, were normal. Paroxetine was progressively tapered until complete discontinuation, while clonazepam 0.5 mg at bedtime was continued.

After 3 months free of paroxetine, the patient reported a gradual increase in anxiety symptoms, with a GAD‐7 score of 10. Agomelatine was initiated at 25 mg at bedtime and increased to 50 mg, leading to a marked reduction in anxiety by week 4 of treatment (GAD‐7 score returned to 0). The prognosis for seizures possibly related to paroxetine was considered favorable. During 24 months of follow‐up, no further seizure episodes were observed. The patient is aware that she experienced two generalized events but does not report significant anxiety about them, as she has no memory of the episodes. Baseline and regular follow‐up LFTs were closely monitored throughout the treatment, and all results remained within normal limits. No adverse effects or abnormal laboratory findings related to agomelatine were observed.

Applying the Naranjo algorithm to assess drug causality yielded a score of 5, indicating a ’Probable’ adverse reaction to high‐dose paroxetine. Agomelatine was a safer and equally effective option for her GAD compared with SSRI treatment. The patient provided written informed consent for publication of her clinical data.

## 4. Discussion

The relationship between seizures and the use of antidepressants has been well documented, although this effect is less common with SSRIs. It has been observed that patients who develop seizures while undergoing antidepressant treatment often have one or more predisposing factors [16]. The two cases exemplify the clinical features, differential diagnosis considerations, therapeutic approach, and prognosis of patients who experienced seizure events temporally associated with antidepressant treatment.

In the first case, the patient had several comorbidities, including type‐I diabetes mellitus, cardiac arrhythmia, and a history of lung tuberculosis. This patient was resistant to several antidepressants and experienced multiple seizure events. The complexity of the case was heightened by polypharmacy; for example, the concurrent use of olanzapine, even at a low dose of 5 mg/day, should be considered a potential confounding factor as atypical antipsychotics are known to lower the seizure threshold. In this patient, agomelatine not only alleviated the depressive symptoms but was also well‐tolerated, with no further seizure events reported after 2 years of observation. In the second case, a patient with GAD experienced two seizures after discontinuing anticonvulsant medication with both high and standard doses of paroxetine. Agomelatine use showed good tolerance and GAD efficacy with no further seizures reported after 2 years of follow‐up.

Numerous studies indicate that patients with major depression face a higher risk of experiencing seizures compared to the general population. While the incidence of unprovoked seizures in the general population is ~56–60 per 100,000 person‐years [7], drug‐naive patients suffering from major depression exhibit a seizure incidence that is 19 times higher [17]. Specifically, seizures are expected to occur in about 1.2% of untreated patients with major depression within a year, compared to just 0.06% in the general population [7].

Epilepsy and other neurological disorders frequently coexist with psychiatric comorbidities, such as affective disorders [18]. Multiple studies have explored the relationship between epilepsy and affective disorders, finding a higher prevalence of epilepsy among patients with conditions like depression and anxiety [3, 19]. The reasons for this association—whether the psychiatric disorders stem from the quality‐of‐life impairment caused by epilepsy or from underlying neurochemical factors—remain unclear. Research indicates a bidirectional relationship between epilepsy and psychiatric disorders [20].

Most antidepressant medications appear to be safe for patients with epilepsy when used at therapeutic doses [3, 6]. According to the 2022 International League Against Epilepsy practice recommendations, SSRIs are usually considered first‐line treatments for depression in patients with epilepsy or elevated seizure risk due to their relatively safe profile compared to older antidepressants [21]. Interestingly, while antidepressants can act as anticonvulsants at lower doses, they may induce seizures when administered at higher doses [22, 23].

The mechanism of action for seizure events associated with the use of antidepressants has been extensively studied, but it remains controversial. The serotonergic and noradrenergic effects of antidepressants’ proconvulsant properties have raised questions among researchers [24]. Jobe and Browning speculate that the convulsions induced by antidepressant therapies may be linked to various factors, including the induction of GABAergic deficiencies, glutamatergic overloads, H1 receptor blockade, the inhibition of G‐protein‐activated inwardly rectifying K + channels (GIRKs), and/or excessive release of brain‐derived neurotrophic factor (BDNF). Additionally, glutamate has been suggested to play a significant role in both depression and the action of antidepressant drugs [25]. It is evident that treatments that increase glutamate neurotransmission could potentially heighten the risk of seizures, and some evidence suggests that certain antidepressants may indeed increase glutamate neurotransmission [26].

Agomelatine has been proposed as a potential treatment option for patients with epilepsy. A retrospective study involving 113 epileptic patients compared the effects of agomelatine with those of escitalopram [27]. The results showed no significant increase in seizure activity for either treatment, with both medications demonstrating similar antidepressant effects. However, the agomelatine group exhibited greater efficacy in treating anxiety and insomnia [27]. Furthermore, in animal models, agomelatine has shown an anticonvulsant effect [28, 29]. This proposed seizure‐protective profile is driven by its dual mechanism. MT1/MT2 agonism exerts well‐documented neuroprotective and antioxidant effects, while its targeted 5‐HT2C antagonism modulates glutamatergic neurotransmission. Together, these properties could prevent excitotoxicity and glutamatergic excess, often involved in antidepressant‐induced seizures [9, 10]

Clinicians encountered challenges in diagnosing seizure episodes associated with antidepressant treatment in both cases presented. In the first case, it was difficult to identify the cause of the multiple seizures due to comorbid conditions, including type 1 diabetes and third‐degree AV block, in a patient with treatment‐resistant depressive disorder. An important differential diagnosis in this case, particularly given the psychiatric comorbidities, medical background, and normal EEGs, is psychogenic non‐epileptic seizures (PNES). However, the presence of severe physical trauma, such as a fracture‐dislocation of the shoulder, along with tongue biting and loss of sphincter control, strongly supports the diagnosis of genuine generalized tonic‐clonic seizures rather than PNES [30].

In the second case, involving a patient with GAD, a seizure emerged 3 weeks after the complete discontinuation of valproic acid. This raised the possibility of a withdrawal seizure. However, evidence suggests that withdrawal features, including recurrent rebound seizures, are largely avoided when antiepileptic drugs are tapered gradually rather than discontinued abruptly [31]. Since valproic acid was tapered gradually and the seizure occurred 3 weeks after it was stopped, withdrawal was deemed a less likely cause. As a result, clinical attention turned to the ongoing high‐dose paroxetine therapy (40 mg/day), which may have lowered the patient’s seizure threshold after the removal of the anticonvulsant medication.

Agomelatine was found to be well‐tolerated and effective in reducing both depressive and anxiety symptoms in both cases, with no seizure activity reported. However, clinicians should remain cautious about its potential hepatotoxic effects. It is crucial to perform mandatory baseline and periodic monitoring of LFTs and other relevant laboratory work, especially in patients with complex medical histories, such as the first case described.

## 5. Conclusion

Among patients at elevated risk for antidepressant‐related seizures, selecting agents with minimal proconvulsant potential is critical. Although agomelatine showed favorable tolerability in the present cases, causal relationships cannot be definitively established from the case reports. Therefore, its use in individuals who experienced seizures associated with SSRIs warrants further systematic investigation to validate these preliminary observations.

## Funding

This research did not receive any specific grant from funding agencies in the public, commercial, or not‐for‐profit sectors.

## Ethics Statement

Written informed consent was obtained from both patients for the publication of this two‐case report and any accompanying clinical information. All clinical data were deidentified to ensure patient anonymity. As this work is a retrospective report of two cases treated in routine clinical practice and does not involve an experimental protocol, formal institutional review board (IRB) approval was waived in accordance with institutional and local guidelines.

## Conflicts of Interest

The authors declare no conflicts of interest.

## Data Availability

The authors confirm that the data can be provided upon request.
